# Adsorption Study of Novel Gemini Cationic Surfactant in Carbonate Reservoir Cores—Influence of Critical Parameters

**DOI:** 10.3390/ma15072527

**Published:** 2022-03-30

**Authors:** Sarmad Khan, Afeez Gbadamosi, Kion Norrman, Xianmin Zhou, Syed Muhammad Shakil Hussain, Shirish Patil, Muhammad Shahzad Kamal

**Affiliations:** 1 Center for Integrative Petroleum Research, King Fahd University of Petroleum & Minerals, Dhahran 31261, Saudi Arabia; sarmad@kfupm.edu.sa (S.K.); kion.norrman@kfupm.edu.sa (K.N.); xzhou@kfupm.edu.sa (X.Z.); shahzadmalik@kfupm.edu.sa (M.S.K.); 2 Department of Petroleum Engineering, King Fahd University of Petroleum & Minerals, Dhahran 31261, Saudi Arabia; a.gbadamosi@kfupm.edu.sa

**Keywords:** adsorption, surfactant, carbonate, gemini, enhanced oil recovery

## Abstract

Surfactant flooding is an enhanced oil recovery method that recovers residual and capillary trapped oil by improving pore-scale displacement efficiency. Low retention of injected chemicals is desired to ensure an economic and cost-effective recovery process. This paper examines the adsorption behavior of a novel gemini cationic surfactant on carbonate cores. The rock cores were characterized using an X-ray diffraction (XRD) spectroscope. In addition, the influence of critical parameters on the dynamic adsorption of the cationic gemini surfactant was studied by injecting the surfactant solution through carbonate cores in a core flooding apparatus until an equilibrium state was achieved. The concentration of surfactant was observed using high performance liquid chromatography. Experimental results showed that an increasing surfactant concentration causes higher retention of the surfactant. Moreover, increasing the flow rate to 0.2 mL/min results in lowering the surfactant retention percentage to 17%. At typical high salinity and high temperature conditions, the cationic gemini surfactant demonstrated low retention (0.42 mg/g-rock) on an Indiana limestone core. This study extends the frontier of knowledge in gemini surfactant applications for enhanced oil recovery.

## 1. Introduction

Despite recent development in alternative sources of energy, crude oil is still a major energy source [1]. About one third of the OOIP (original oil in place) can be obtained using primary and secondary oil recovery techniques [2]. To meet the ever-increasing energy demand, companies prefer to extract more oil from the existing oil wells than to invest a high capital outlay on the exploration and production of new wells [3]. To extract the remaining hydrocarbon, a tertiary recovery technique, i.e., enhanced oil recovery (EOR) technique, is applied. Among various EOR methods (thermal, microbial, gas injection), chemical EOR is attractive for specific reservoirs [4].

Surfactant flooding, a chemical EOR method, is used to recover oil from reservoirs through minimizing the interfacial tension and altering the wettability of the porous media [5]. However, the selection of surfactants for chemical EOR plays a crucial role in its efficiency [6]. The adsorption of surfactants in porous media is one of the challenges in surfactant flooding [7,8]. This phenomenon occurs when the surfactant molecules gather at the liquid–solid interface due to rock–fluid interactions [9]. Oil reservoirs can be classified as carbonate or sandstone. The carbonate reservoirs are mainly calcite (alternate layers of Ca^2+^ and CO_3_^2–^) and/or dolomite (alternate layers of Ca^2+^, Mg^2+^, and CO_3_^2–^). The carbonate rocks are considered as positively charged. Therefore, cationic surfactants are preferred to minimize the adsorption due to the charge repulsion [10]. On the contrary, sandstone reservoirs are considered as negatively charged due to a large amount of quartz (SiO_2_, silica). Anionic surfactants are preferred in sandstone reservoirs to reduce the adsorption, once again, because of the static repulsion [11]. 

Several mechanisms are involved in surfactant adsorption, including electrostatic attraction, van der Waals interaction, hydrogen/covalent bonding, polarization, etc. [12,13]. High adsorption of surfactant implies a loss of injected chemicals and lowers the concentration of the surfactant in chemical slugs. The fundamental job of the surfactant is to lower the interfacial tension (IFT) up to 10^–3^ mN/m and/or to alter the wettability of the rock towards water-wetting conditions [14]. However, in the case of adsorption, the surfactants not only lose their ability to minimize IFT and change the wettability of the rock, but also reduce the feasibility of the project economically. The use of several surfactants has been evaluated for the field application of oil recovery [14,15]. This includes nonionic, cationic, anionic, and zwitterionic surfactants. More recently, the use of natural, viscoelastic, polymeric, and gemini surfactants has received prodigious attention [16,17,18,19,20,21,22].

Gemini surfactants have received a lot of attention lately due to their excellent properties, such as high salt tolerance, strong mono and divalent ions resistance, low surface/interface values, and high heat stability [23]. Gemini surfactants with two lipophilic tails and two lipophobic heads show lower adsorption densities compared to the conventional surfactants containing one tail and one head group [24]. Páhi and co-workers [25] prepared a range of gemini surfactants and compared the adsorption behavior with the conventional monomeric counterpart on sandstone. It was observed that the gemini surfactant exhibited the lowest equilibrium adsorption density value as compared to the conventional surfactants. Chen et al. [26] studied the role of high salinity conditions on gemini surfactants and found the lowest adsorption density values in the presence of salts. Zhao et al. synthesized cationic gemini surfactants containing different hydrophobic tail lengths and studied the role of the hydrophobic tail on adsorption capacities and adsorption morphologies [27]. It was found that the gemini cationic surfactant can prevent the acid–rock reaction by creating a monolayer on the rock surface. Besides, the adsorption morphology can be controlled by varying the surfactant concentration. Yang and co-workers studied the role of saturated and unsaturated hydrophobic tails and spacer groups of gemini cationic surfactants in terms of adsorption and aggregation behavior [28]. They concluded that the surfactant with an unsaturated hydrophobic tail exhibits better surface activity, and that the adsorption phenomenon can be controlled through a mixed diffusion–kinetic adsorption mechanism.

Cationic surfactants are found in many household and cleaning products which end up in the aquatic environment. The insertion of a cleavable bond in the spacer of gemini surfactants is important from a biodegradation point of view. Gemini cationic surfactants having an amide group exhibit low toxicity, good biodegradability, and are environmentally benign. The amide group in the chemical structure of surfactants is considered a biodegradable connection suitable for the formation of eco-friendly gemini cationic surfactants [29]. Similarly, a readily cleavable ester bond in the spacer group of gemini surfactants is advantageous to form environmentally friendly surfactants [30]. 

Previous studies on the adsorption behavior of gemini surfactants were either evaluated on sandstone rocks or by using a static adsorption process. Moreover, the influence of critical parameters on the adsorption behavior of gemini surfactants in carbonates remains obscure in the literature. Herein, the adsorption properties of a novel synthesized gemini cationic surfactant on carbonate rock were studied. Firstly, the dynamic adsorption properties of the surfactant on carbonate cores were examined using a high-pressure high-temperature (HPHT) core flooding apparatus. The surfactant concentration before and after the core flooding process was monitored using high pressure liquid chromatography (HPLC) to determine the surfactant retention. Finally, the effect of critical parameters, such as varying conditions of temperature and salinity, flow rate, and surfactant concentration, on the adsorption property of the cationic gemini surfactant were determined.

## 2. Materials and Methods

### 2.1. Surfactant

The following materials were purchased from Sigma Aldrich: aluminium oxide (Al_2_O_3_), 1,12-dibromododecane (99%), NaF (99%), 3-dimethylamino-1-propylamine (99%), glycolic acid ethoxylate lauryl ether (average $m_{n}$ ~ 690). NaCl, CaCl_2_, Na_2_SO_4_, MgCl_2_, and NaHCO_3_, were all used for formulating the brine compositions and obtained from Panreac. Indiana limestone carbonate core samples were used.

### 2.2. Rock Samples

The study utilized Indiana limestone (depicted ILLZ) cores, which were cleaned with methanol and toluene in a Soxhlet extractor and thereafter dried out using an oven at 60 °C. After drying, the porosity and permeability of the cores were determined using an automated porosimeter and Permeameter 608 from Coretest Inc. The limestone core sample was characterized using X-ray diffraction (XRD) spectroscope from Pananalytical, Malvern, United Kingdom. Subsequently, the cores were saturated with either the formation water or with deionized (DI) water based on the experiment.

### 2.3. Brine Preparation

There were two kinds of experiments performed in this study. Firstly, formation water was used to saturate the core, and seawater was used for primary flooding. The salinity of the formation water and seawater was 241,688 ppm and 67,779 ppm, respectively. In addition, de-ionized water was used both for saturation and primary flooding.

### 2.4. Preparation of the Cationic Gemini Surfactant

**Figure 1:**
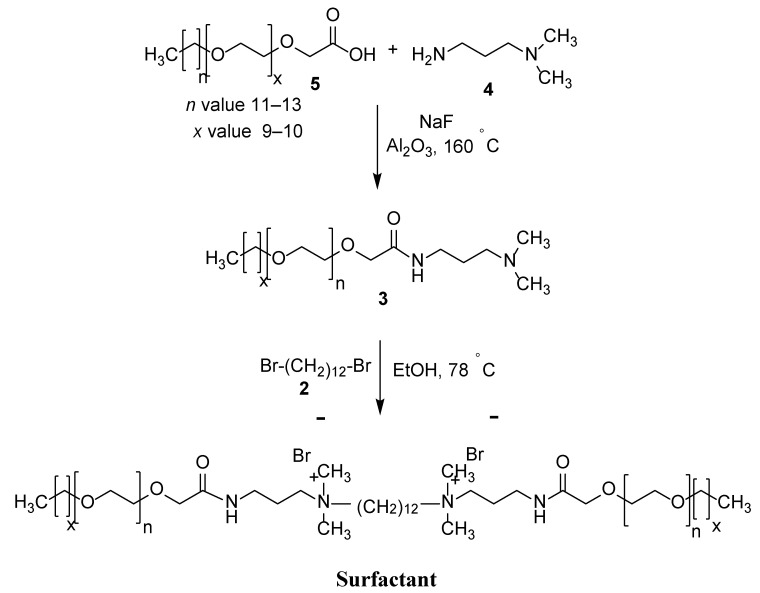
Preparation method of surfactant.

Figure 1 depicts the procedure for the synthesis of the cationic Gemini surfactant with ethylene oxide (EO) units. Using a 250 mL round bottom flask, 8.89 g of 86.96 mmol 3-dimethylamino-1-propylamine (**4**) were reacted with 30 g of 43.48 mmol glycolic acid ethoxylate lauryl ether (**5**) in the presence of sodium fluoride. The resultant mixture was stirred for up to six hours under argon at 160 °C. Subsequently, the aqueous phase in the mixture was captured by Al_2_O_3_ (alumina). Thereafter, the mixture was reacted with an additional 6.66 g of 65.22 mmol 3-dimethylamino-1-propylamine (**4**) and left for an additional four hours. The unreacted mixture was left to evaporate, and the remaining residue was purified using the filtration method to achieve the intermediate (**3**). Finally, 10.0 g of 12.97 mmol of the derived intermediate was reacted with 1.84 g of 5.62 mmol 1,12-dibromododecane (**2**) for 48 h in 5 mL of ethanol. The Gemini surfactant was obtained by subjecting the reaction mixture to flash column chromatography followed by vacuum drying.

### 2.5. Adsorption Study

**Figure 2:**
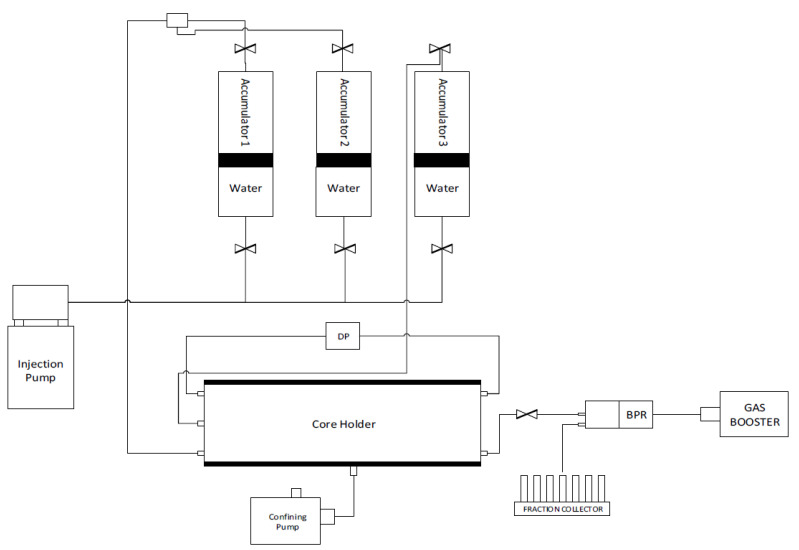
Schematic diagram of the Core flooding apparatus.

The dynamic adsorption behavior of the surfactant in question was conducted in the HPHT DAS-100 Data Acquisition System from CoreLab (see Figure 2). The utilized fluid properties are given in Table 1. The equipment consists of an oven, three accumulators, a core holder, injection pump, confining pressure system, transducers, injection pump, back pressure regulator, fraction collector, and a digital data recording system. Various automatic and manual valves were mounted in the system to control the flow of fluids. Saturated cores were loaded into the core holder and confining pressure of 4500 psi was gradually applied. The backpressure regulator was set up at 3200 psi and the saturating fluid was injected at 1 mL/min until the pore pressure reached the back pressure, which usually took a few pore volumes (PV) of injection. For experiments at high temperatures, the system was heated and stabilized at 102 °C. Otherwise, the primary flood directly proceeded. After primary flooding, surfactant injection was started at a pre-decided rate (0.05, 0.1, or 0.02 mL/min) and about 10–15 pore volumes were injected. The effluent from the core was collected using an automatic fraction collector. The volume per vial was kept at 2 mL in the initial experiments but was changed to 4 mL in later experiments. The primary idea was to reach an equilibrium value such that the surfactant concentration of the effluent becomes equal to the concentration of the fresh surfactant solution. The differential pressure across the core was recorded in this phase so that the permeability to the surfactant solution could be determined. While maintaining the same flow rate as that of surfactant flood, either seawater or de-ionized water (depending upon the type of the experiment) was re-injected in the core to displace the surfactant solution. The effluent was collected and measured at the fraction collector. About 10 pore volumes were injected to ensure that the surfactant concentration of the effluent dropped to zero. After this, the flow rate was varied a few times to obtain the final permeability of the core at three different rates, after which the experiments were terminated. The pore pressure and confining pressures were released, and the oven turned off. The core plugs were unloaded, and the lines were flushed with formation water or de-ionized water.

### 2.6. Determination of the Magnitude of Dynamic Adsorption and Desorption

The surfactant concentration in the effluent, C_s_, was determined from the HPLC data by the following procedure. Chromatographic separations were carried out on an Acclaim™ Surfactant Plus HPLC Column from Thermo Scientific™ (150 mm × 4.6 mm, 3.0 µm porosity, Lot No. 01834010) with the applied volume of 1.0 µL, 1.0 mL/min rate of flow, and column temperature of 30 °C. The mobile phase was composed of 0.1 M ammonium formate (NH_4_HCO_2_) at pH 4.3 and acetonitrile. Samples were analyzed by employing the following gradient program: 100% ammonium formate with a linear gradient to 100% acetonitrile up to 10 min, before a linear gradient up to 100% ammonium formate over 1 min with 2.0 mL/min rate of flow. A high-performance liquid chromatography (HPLC) system from Agilent (1290 Infinity II) connected to an evaporative light scattering detector (ELSD, 1260 Infinity II) was employed. Data evaluation was performed using the OpenLAB CDS ChemStation Edition (Version 2.17.29) software package. The ELSD peak areas were plotted against known concentrations that produced the expected non-linear (ELSD is a non-linear detector) calibration curve, which was used to obtain the effluent concentrations from the adsorption experiments. The surfactant concentration obtained was then used to calculate the adsorption and desorption values according to Equation (1).

(1) $$q=\frac{C_{i}-C_{f}}{1000m}\times V_{s}\;\;$$

where *q* is the surfactant retention on rock surface (mg/g-rock), $V_{s}$ is the total volume of original bulk solution (mL), $C_{i}$ is the initial concentration of surfactant (mg/L), $C_{f}$ is the final concentration of surfactant (mg/L), and m is the mass of the core (g).

## 3. Results and Discussion

### 3.1. XRD Characterization of the Rock

Table 2 presents the XRD results of the Indiana limestone used in this study. The limestone has a high degree of calcite, thus demonstrating its carbonate core properties. Table 3 presents the reservoir rock properties of the cores. The cores have relatively low permeabilities, which are characteristic of carbonate cores [31].

### 3.2. Adsorption Equilibrium Studies

**Figure 3:**
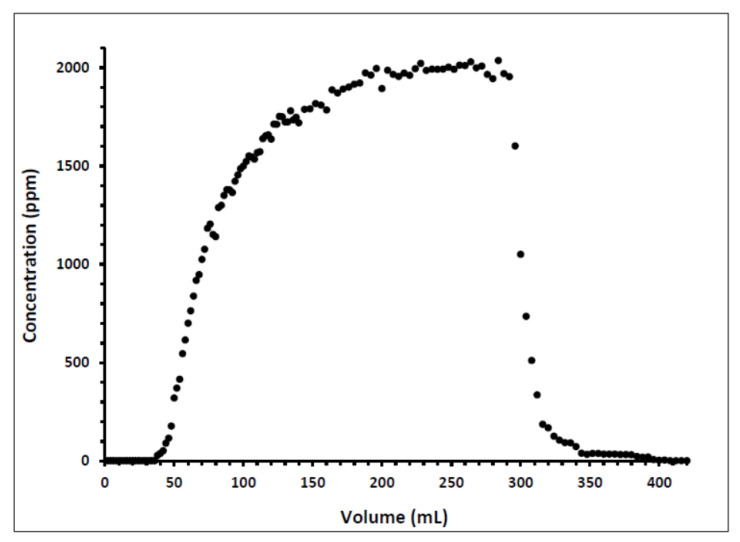
Concentration profile of ILLZ-7 effluent.

**Figure 4:**
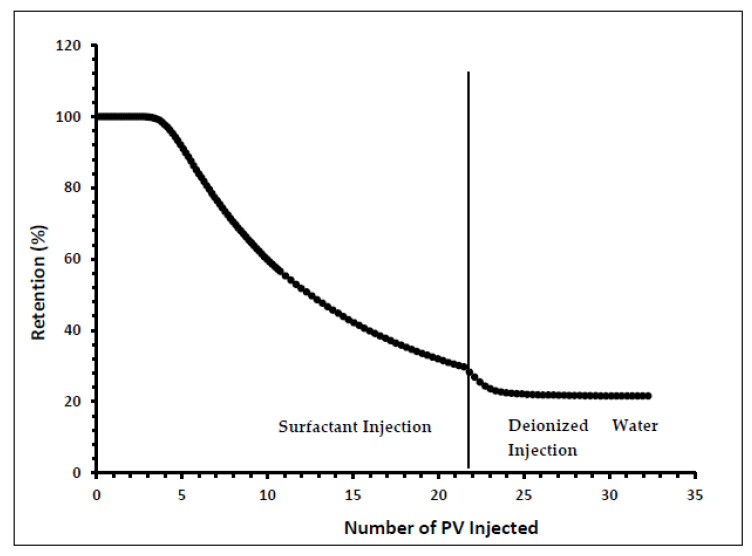
Retention percentage profile for ILLZ-7.

**Figure 5:**
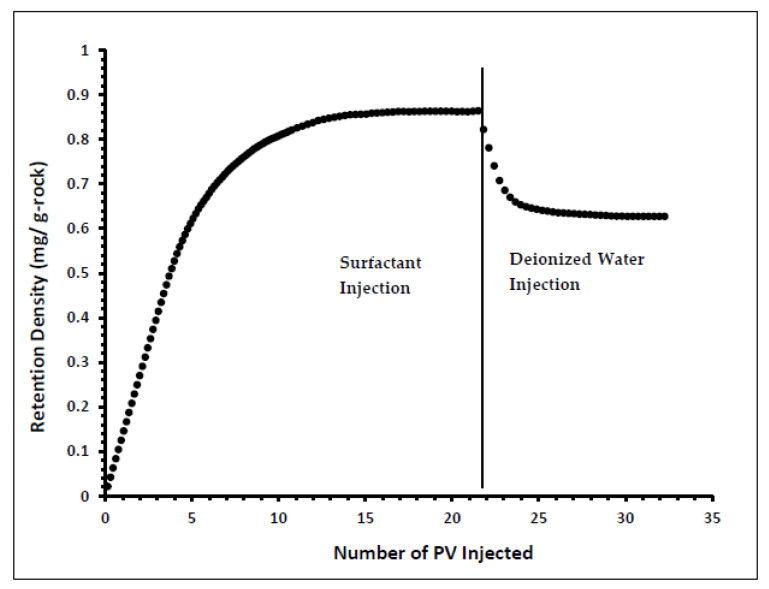
Retention Density Profile of ILLZ-7.

Indiana limestone core (ILLZ-7) saturated with deionized water was used. A surfactant with a concentration of 2000 ppm was injected into the core at room temperature. The flow rate used for this experiment was 0.1 mL/min. The HPLC analysis (Figure 3) showed a maximum reading of about ~2034 ppm, which corresponds to all the surfactant flowing out of the core at the end of surfactant injection. It was also inferred that after introducing 11 PV deionized water, the core retained 22% of the adsorbed surfactant on the walls of the pores while the surfactant retention density was 0.63 mg/g-rock (see Figure 4 and Figure 5). The surfactant retention may be attributed to the polyoxyethylene chain present in the surfactant. A strong electrostatic interaction presumably occurs between the oxygen in polyethylene oxide of the surfactant and the Ca^2+^ ions of the core surface, which causes packing of the surfactant at the rock–liquid interface, and thus its adsorption [32]. Moreover, the larger surface area that the gemini surfactants occupy on the core surface due to the long length of their hydrophobic chains may have contributed to the adsorption of the surfactant at the solid–liquid interface [33].

#### 3.2.1. Effect of Surfactant Concentration on Dynamic Adsorption

**Figure 6:**
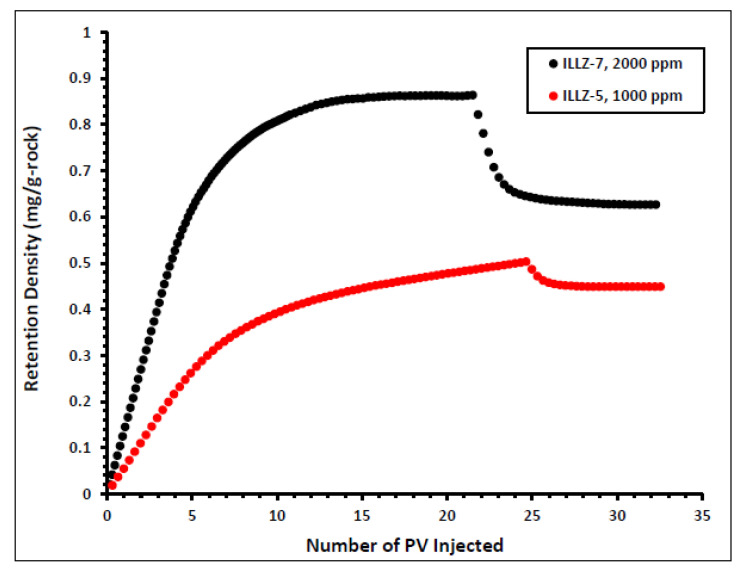
Effect of surfactant concentration on retention density.

The impact of varying the concentration of the injected surfactant on the adsorption properties of Gemini cationic surfactant was studied and the results are shown in Figure 6. The surfactant concentration was changed from 2000 ppm on ILLZ-7 cores to 1000 ppm in the experiment on core ILLZ-5. For 1000 ppm surfactant concentration, the retention density of the surfactant is approximately 0.45 mg/g-rock. This implies that a lower concentration of injected surfactant yields lower retention density. The high retention density recorded at high surfactant concentration may have resulted from lateral interactions between the surfactant molecules, which cause the formation of aggregate at the surface of the reservoir core [14,34,35].

#### 3.2.2. Effect of High Temperature and High Salinity on Surfactant Behavior

**Figure 7:**
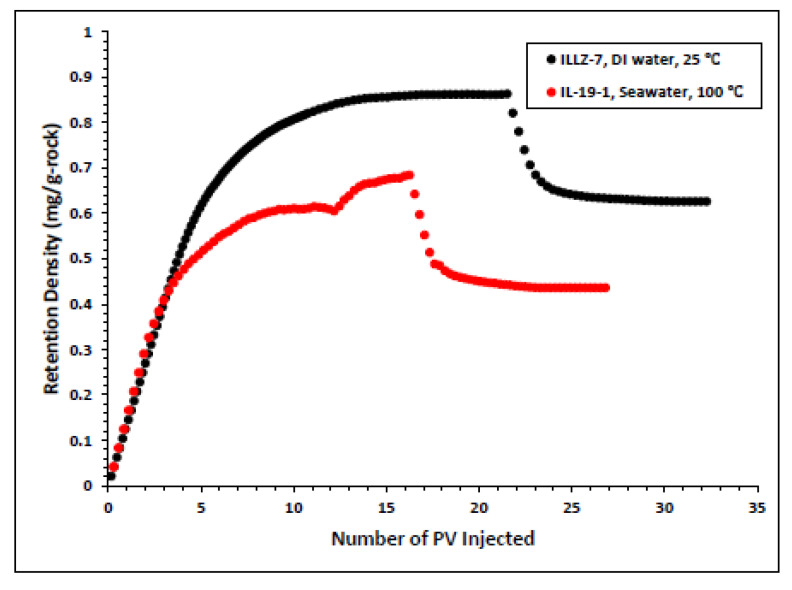
Surfactant density profile at ambient and HTHS condition.

**Figure 8:**
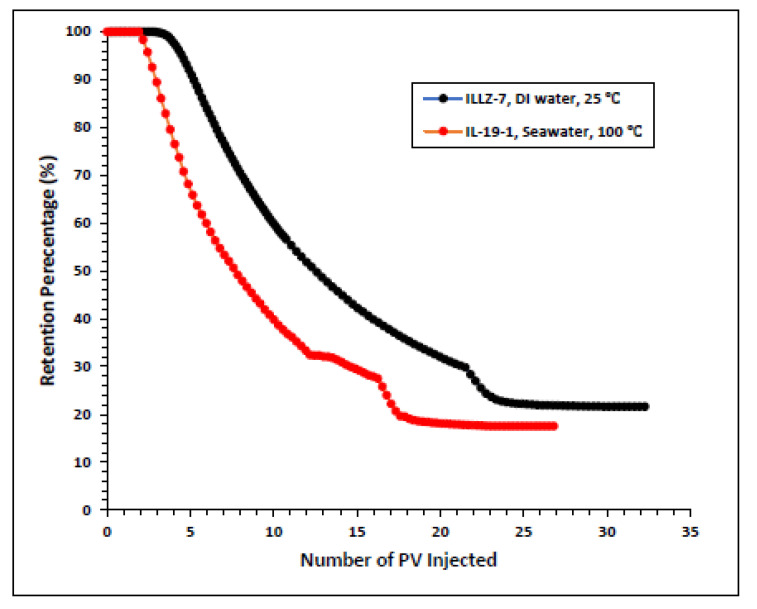
Comparison of surfactant retention percentage profile of simplified and real conditions.

**Figure 9:**
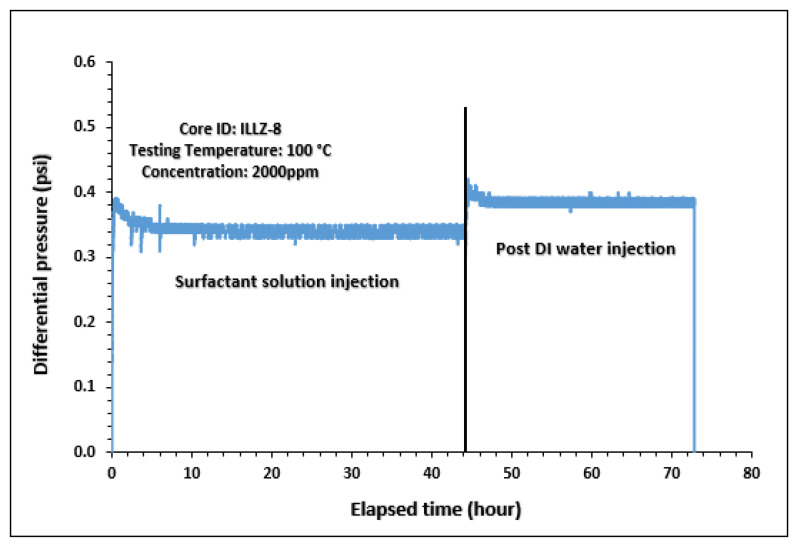
Differential pressure during surfactant flooding and post water injection.

The surfactant behavior at typical high temperature and high salinity (HTHS) reservoir conditions was investigated. To mimic the reservoir condition, the core was saturated with formation water and the temperature was kept at 102 °C. The maximum concentration detected on HPLC was ~2167 ppm, from which it is inferred that all the surfactant was flowing out towards the end of the surfactant injection phase. Figure 7 and Figure 8 depict the retention density profile and retention percentage of the gemini cationic surfactant, respectively. As compared to a similar experiment with deionized water as the saturating fluid, it was found that the retention density, as well as the retention percentage of the surfactant, decreased with increased temperature and salinity. This further confirms that the surfactant was stable at HTHS conditions. Moreover, the lower retention density of the surfactant at HTHS conditions may be adduced to the encapsulation of the cations by the long ether chain of the surfactant, hence inhibiting the retention at the rock surface [36]. Additionally, the increase in salinity causes the positive charge on the carbonate surface to increase, which causes repulsion between the limestone and the positively charged cationic gemini surfactant. The pressure drop profile of the surfactant retention behavior on Indiana limestone at typical reservoir conditions is depicted in Figure 9. The differential pressure during post-water injection was slightly higher than the differential pressure during surfactant flooding. This confirms the retention of surfactants on the surface of the rock pores. Moreover, the post-injection deionized water may have caused the mineral dissolution of the carbonate core [37].

#### 3.2.3. Effect of Flow Rate

**Figure 10:**
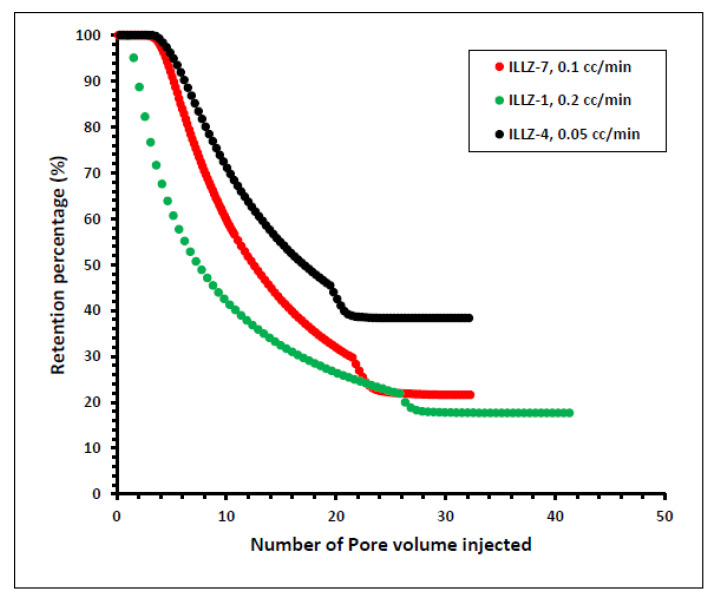
Effect of flow rate on surfactant retention percentage.

In another experiment on ILLZ-1, the rate of flow was enhanced from 0.1 mL/min to 0.2 mL/min while the other parameters remained the same as that of ILLZ-7. A maximum of ~1929 ppm of surfactant was detected on HPLC while the retention was slightly reduced to 17% owing to the high rate of flow. To further determine the impact of the rate of flow on this phenomenon, the rate of flow was reduced to 0.05 mL/min in the experiment on core ILLZ-4 while the concentration remained at 2000 ppm and the temperature at room conditions. It was found that the retention percentage increased to 38.5%, as shown in Figure 10. The low retention of the surfactant at a high flow rate can be attributed to the lower contact time between the surfactant molecules and the Indiana limestone core surface. At a low rate of flow, the time of contact between the hydrophobic moieties of the surfactant and the interstices of the rock surface is high. Hence, the inter-particle repulsive force between the cationic gemini surfactant and the limestone core surface is decreased, and consequently, the retention is high [38,39,40].

## 4. Conclusions

This study investigates the dynamic adsorption properties of novel synthesized cationic gemini surfactants on Indiana limestone cores. Experimental results demonstrate that increasing the surfactant concentration results in a higher retention of the surfactant on limestone. Contrariwise, increasing the flow rate has an inverse effect on the retention of the surfactant, which means that a higher percentage of the injected surfactant is retained at lower flow rates. At typical reservoir conditions (formation water salinity = 241,688 ppm, seawater salinity = 67,779 ppm, and temperature = 102 °C), the cationic gemini surfactant exhibited moderate retention behavior (0.42 mg/g-rock) in limestone cores, which is considered a favorable property for EOR processes. The differential pressure profile confirms the retention of surfactant as the differential pressure after surfactant flooding was higher than the differential pressure during the surfactant flooding process. Overall, the retention density of the surfactant is less than 1 mg/g-rock, and hence deemed suitable for EOR.

## Figures and Tables

**Table 1:** Properties of the fluids used.

Fluids	Concentration	Temperature, 25 °C		Temperature, 100 °C	
	ppm	Density (g/mL)	Viscosity (cP)	Density (g/mL)	Viscosity (cP)
**Formation Water**	-	1.1462	1.4500	1.1083	0.4760
**Seawater**	-	1.0385	1.3800	1.0152	0.3270
**DI Water**	-	0.9968	0.89	0.9565	0.308
**Surfactant**	500	0.9959	0.8729	0.8724	0.2199
	1000	0.9961	0.8868	0.8949	0.2337
	2000	0.9964	0.8979	0.9545	0.2625

**Table 2:** XRD result of core sample.

Mineral	%
Calcite	98.5
Quartz	0.2
Dolomite	0.3
Illite	0.3
Kaolinite	0.1
Alunite	0.5
Anorthite	0
Halite	0.1

**Table 3:** Rock properties.

Sample ID (ft)	Dry Weight (gm)	Average Length (cm)	Average Diameter (cm)	Porosity (%)	Permeability (mD)
ILLZ-1	183.97	7.586	3.77	19.37	20.38
ILLZ-4	192.92	7.608	3.78	15.19	17.7
ILLZ-5	192.95	7.617	3.78	15.05	13.4
ILLZ-7	192.95	7.614	3.78	15.24	18.12
ILLZ-8	193.42	7.613	3.78	15.02	22.47
IL-19-1	190.67	7.692	3.78	17.04	35

## Data Availability

Not applicable.

## References

[1] Bashir A., Sharifi Haddad A., Rafati R. A review of fluid displacement mechanisms in surfactant-based chemical enhanced oil recovery processes: Analyses of key influencing factors. Pet. Sci..

[2] Tavakkoli O., Kamyab H., Shariati M., Mustafa Mohamed A., Junin R. (2022). Effect of nanoparticles on the performance of polymer/surfactant flooding for enhanced oil recovery: A review. Fuel.

[3] Massarweh O., Abushaikha A.S. (2020). The use of surfactants in enhanced oil recovery: A review of recent advances. Energy Rep..

[4] Gbadamosi A., Junin R., Manan M., Agi A., Oseh J., Samsuri A. (2019). Nanotechnology Application in Chemical Enhanced Oil Recovery: Current Opinion and Recent Advances. Enhanced Oil Recovery Processes—New Technologies.

[5] Abbas A.H., Sulaiman W.R.W., Jaafar M.Z., Gbadamosi A.O., Ebrahimi S.S., Elrufai A. (2020). Numerical study for continuous surfactant flooding considering adsorption in heterogeneous reservoir. J. King Saud Univ. Eng. Sci..

[6] Chen W., Schechter D.S. (2021). Surfactant selection for enhanced oil recovery based on surfactant molecular structure in unconventional liquid reservoirs. J. Pet. Sci. Eng..

[7] Pal N., Hoteit H., Mandal A. (2021). Structural aspects, mechanisms and emerging prospects of Gemini surfactant-based alternative Enhanced Oil Recovery technology: A review. J. Mol. Liq..

[8] Tumba J., Agi A., Gbadamosi A., Junin R., Abbas A., Rajaei K., Gbonhinbor J. Lignin as a Potential Additive for Minimizing Surfactant Adsorption On Clay Minerals In Different Electrolyte Concentration. Proceedings of the SPE Nigeria Annual International Conference and Exhibition.

[9] Liu Z., Zhao G., Brewer M., Lv Q., Sudhölter E.J.R. (2021). Comprehensive review on surfactant adsorption on mineral surfaces in chemical enhanced oil recovery. Adv. Colloid Interface Sci..

[10] Standnes D.C., Austad T. (2003). Wettability alteration in carbonates: Interaction between cationic surfactant and carboxylates as a key factor in wettability alteration from oil-wet to water-wet conditions. Colloids Surf. A Physicochem. Eng. Asp..

[11] Gbadamosi A.O., Junin R., Manan M.A., Agi A., Yusuff A.S. (2019). An overview of chemical enhanced oil recovery: Recent advances and prospects. Int. Nano Lett..

[12] Lv W., Bazin B., Ma D., Liu Q., Han D., Wu K. (2011). Undefined Static and Dynamic Adsorption of Anionic and Amphoteric Surfactants with and without the Presence of Alkali.

[13] Kalam S., Abu-Khamsin S.A., Kamal M.S., Patil S. (2021). A review on surfactant retention on rocks: Mechanisms, measurements, and influencing factors. Fuel.

[14] Kamal M.S., Hussein I.A., Sultan A.S. (2017). Review on Surfactant Flooding: Phase Behavior, Retention, IFT, and Field Applications. Energy Fuels.

[15] Kamal M.S., Sultan A.S., Al-Mubaiyedh U.A., Hussein I.A. (2015). Review on polymer flooding: Rheology, adsorption, stability, and field applications of various polymer systems. Polym. Rev..

[16] Shehzad F., Hussain S.M.S., Adewunmi A.A., Mahboob A., Murtaza M., Kamal M.S. (2021). Magnetic surfactants: A review of recent progress in synthesis and applications. Adv. Colloid Interface Sci..

[17] Hussain S.M.S., Adewunmi A.A., Mahboob A., Murtaza M., Zhou X., Kamal M.S. (2022). Fluorinated surfactants: A review on recent progress on synthesis and oilfield applications. Adv. Colloid Interface Sci..

[18] Hussain S.M.S., Mahboob A., Kamal M.S. (2020). Synthesis and Evaluation of Zwitterionic Surfactants Bearing Benzene Ring in the Hydrophobic Tail. Materials.

[19] Hussain S.M.S., Mahboob A., Kamal M.S. (2020). Poly (oxyethylene)-amidoamine based gemini cationic surfactants for oilfield applications: Effect of hydrophilicity of spacer group. Materials.

[20] Villa C., Baldassari S., Chillura Martino D.F., Spinella A., Caponetti E. (2013). Green Synthesis, Molecular Characterization and Associative Behavior of Some Gemini Surfactants without a Spacer Group. Materials.

[21] Bin-Hudayb N.S., Badr E.E., Hegazy M.A. (2020). Adsorption and corrosion performance of new cationic gemini surfactants derivatives of fatty amido ethyl aminium chloride with ester spacer for mild steel in acidic solutions. Materials.

[22] Shekhovtsova S., Korolev E. (2022). Interfacial Phenomena at the Interface in the System «Carbon Primary Materials-Water Solutions of Surfactants» for Cement Materials. Materials.

[23] Deng X., Shahzad Kamal M., Patil S., Muhammad Shakil Hussain S., Zhou X., Mahmoud M. (2021). Wettability alteration of locally synthesized cationic gemini surfactants on carbonate rock. J. Mol. Liq..

[24] Kamal M.S. (2016). A Review of Gemini Surfactants: Potential Application in Enhanced Oil Recovery. J. Surfactants Deterg..

[25] Páhi A.B., Király Z., Mastalir Á., Dudás J., Puskás S., Vágó Á. (2008). Thermodynamics of micelle formation of the counterion coupled gemini surfactant bis(4-(2-dodecyl)benzenesuifonate)-jeffamine salt and its dynamic adsorption on sandstone. J. Phys. Chem. B.

[26] Chen Z., Mi H., Liu X., Xia K., Ge F., Li X., Zhang X. (2019). Preparation and Characterization of an Anionic Gemini Surfactant for Enhanced Oil Recovery in a Hypersaline Reservoir. J. Surfactants Deterg..

[27] Zhao F., Wang S., Shen X., Guo J., Liu Y. (2019). Study on mechanism of Gemini surfactant inhibiting acid rock reaction rate. Colloids Surf. A Physicochem. Eng. Asp..

[28] Yang W., Cao Y., Wang Y., Ju H., Jiang Y., Geng T. (2021). Effects of unsaturated double bonds on adsorption and aggregation behaviors of amide-based cationic Gemini surfactants. Colloids Surf. A Physicochem. Eng. Asp..

[29] Taleb K., Mohamed-Benkada M., Benhamed N., Saidi-Besbes S., Grohens Y., Derdour A. (2017). Benzene ring containing cationic gemini surfactants: Synthesis, surface properties and antibacterial activity. J. Mol. Liq..

[30] Tehrani-Bagha A.R., Holmberg K., van Ginkel C.G., Kean M. (2015). Cationic gemini surfactants with cleavable spacer: Chemical hydrolysis, biodegradation, and toxicity. J. Colloid Interface Sci..

[31] Mukhametdinova A., Kazak A., Karamov T., Bogdanovich N., Serkin M., Melekhin S., Cheremisin A. (2020). Reservoir Properties of Low-Permeable Carbonate Rocks: Experimental Features. Energies.

[32] Xu X., Zhao Y., Lai Q., Hao Y. (2011). Effect of polyethylene glycol on phase and morphology of calcium carbonate. J. Appl. Polym. Sci..

[33] Rosen M.J., Li F. (2001). The adsorption of gemini and conventional surfactants onto some soil solids and the removal of 2-naphthol by the soil surfaces. J. Colloid Interface Sci..

[34] Isaac O.T., Pu H., Oni B.A., Samson F.A. (2022). Surfactants employed in conventional and unconventional reservoirs for enhanced oil recovery—A review. Energy Rep..

[35] Belhaj A.F., Elraies K.A., Mahmood S.M., Zulkifli N.N., Akbari S., Hussien O.S.E. (2020). The effect of surfactant concentration, salinity, temperature, and pH on surfactant adsorption for chemical enhanced oil recovery: A review. J. Pet. Explor. Prod. Technol..

[36] Hussain S.M.S., Kamal M.S., Solling T., Murtaza M., Fogang L.T. (2019). Surface and thermal properties of synthesized cationic poly(ethylene oxide) gemini surfactants: The role of the spacer. RSC Adv..

[37] Al-Bayati A., Karunarathne C.I., Al Jehani A.S., Al-Yaseri A.Z., Keshavarz A., Iglauer S. (2022). Wettability Alteration during Low-Salinity Water Flooding. Energy Fuels.

[38] Ahmadi M.A., Shadizadeh S.R. (2018). Spotlight on the New Natural Surfactant Flooding in Carbonate Rock Samples in Low Salinity Condition. Sci. Rep..

[39] Rodriguez A. (1992). Surface Charge Behavior and Adsorption of Surfactants on Carbonate Rocks, King Fahd.

[40] Bae J.H., Petrick C.B. (1977). Adsorption/Retention of Petroleum Sulfonates in Berea Cores. Soc. Pet. Eng. J..

